# Acute exposure to mercury drives changes in gene expression in *Drosophila melanogaster*

**DOI:** 10.1186/s13104-024-06945-y

**Published:** 2024-09-30

**Authors:** Brian J. Sanderson, Dylan J. Sims-West, Stuart J. Macdonald

**Affiliations:** 1 Department of Molecular Biosciences, University of Kansas, Lawrence, KS 66045 USA; 2 Center for Reproductive Evolution, Department of Biology, Syracuse University, Syracuse, NY 13244 USA

**Keywords:** *Drosophila melanogaster*, RNA-seq, Mercury

## Abstract

**Objective:**

We quantified the effect of acute exposure to a high dosage of inorganic mercury on gene expression in *Drosophila melanogaster* using RNA-sequencing of whole adult females.

**Results:**

We found 119 genes with higher gene expression following treatment (including all 5 *Drosophila* metallothionine genes and a number of heat shock protein genes), and 31 with lower expression (several of which are involved in egg formation). Our results highlight biological processes and genetic pathways impacted by exposure to this toxic metal, and provide motivation for future studies to understand the genetic basis of response to mercury.

**Supplementary Information:**

The online version contains supplementary material available at 10.1186/s13104-024-06945-y.

## Introduction

Mercury exposure can occur through contamination of water, air and food, as well as through some household items, and such exposure can lead to a variety of pathologies [1]. The effects of mercury exposure can vary between life history stages, and because mercury exists in both organic and inorganic forms, its toxic effects are also dependent on the form encountered [2]. To help understand the impact of mercury on cellular function, past studies have characterized the impacts of mercury exposure on gene expression in several animal species, including organic methylmercury exposure on mouse pups [3, 4], inorganic mercury chloride in zebrafish [5, 6], and both organic and inorganic mercury in *C. elegans* [2, 7].

*Drosophila melanogaster* is a powerful model for understanding the impact of toxicant exposure [8], and because sets of genotyped inbred strains are available [9, 10], the *Drosophila* model can ultimately enable characterization of the genetic basis of variation in toxicant response [11–13]. Describing the regulatory changes driven by toxicant exposure is key to understanding the physiological response to stress, and work in *Drosophila* has shown that larval exposure to methylmercury [14, 15] and adult exposure to inorganic mercury [16] impact gene expression. Here, we sought to expand this understanding of the impact of mercury exposure on gene expression in *Drosophila* by employing a transcriptome-wide RNA sequencing approach (*versus* employing qRT-PCR on a focused set of genes [14, 16] or expression arrays [15]), examining flies following a high dosage, acute exposure to inorganic mercury. By employing several different strains from a large panel of inbred lines—the *Drosophila* Synthetic Population Resource [DSPR; 10]—we aimed to gain some insight into the mercury-induced expression response in this panel, and facilitate future genetic dissection of variation in the response to mercury toxicity via QTL (Quantitative Trait Locus) mapping, as has been executed for other traits [e.g. 11].

We exposed adult females from 7 DSPR strains to media containing either inorganic mercury(II) chloride (HgCl_2_) or a water control for six hours, extracted RNA from flash-frozen whole animals, and prepared and sequenced short-read mRNA sequencing libraries. An analysis that pooled together all strains identified 119 genes that were up-regulated and 31 genes that were down-regulated in response to this acute inorganic mercury exposure, and collectively these gene sets were enriched for a number of biological processes.

## Materials and methods

### *Drosophila* strains

The 7 strains tested—22033, 22112, 22114, 22117, 22127, 22217, 22238—are part of the DSPR “population B” collection of Recombinant Inbred Lines (RILs). Briefly, the DSPR was founded by intercrossing 8 highly-inbred founder lines, allowing the synthetic population to mix/recombine for 50 generations, and then several hundred RILs were derived by 25 generations of sibling mating [10]. The target strains for this study were chosen arbitrarily to represent a fraction of the variation existing in the panel.

### Rearing experimental animals

Adult flies from each strain were allowed to lay eggs in vials for 2 days before being cleared. Nine days after vial set up any emerged adults were removed, and two days later all 0–2 day old animals were moved to fresh media vials. Mixed sex groups of flies were aged for 2 additional days, and we collected 2–4 day old females via CO_2_ anesthesia, generating 2 vials of 10 females per RIL. These females are likely mated given they were held with males for 48 h, but mating status was not confirmed, as is common practice in the *Drosophila* community. Test female flies were given 1 day to recover from any effects of anesthesia prior to mercury exposure.

### Mercury exposure assay

We exposed sets of 10 female flies to a 625 µM solution of inorganic mercury(II) chloride, HgCl_2_ (Millipore Sigma, 215465, CAS number 7487-94-7), or a water control, in exposure chambers. Each chamber consisted of a standard, narrow polystyrene fly vial (Fisher Scientific, AS515), and a polyethylene flanged plastic cap (MOCAP, FCS13/16NA1) taped to it via narrow masking tape (ULINE, S-3049). The cap contained 1/8th of a teaspoon of Formula 4–24 Instant *Drosophila* Medium (Carolina, 173200) that was finely-ground by using a food processor, and reconstituted with 0.9-ml of either mercury solution or water. Adult females (3–5 days old) from all 7 RILs were enclosed in chambers for approximately 6 h, starting at 1-h after lights on.

### Rearing and testing environment

All rearing/testing was executed in an incubator held at 25C, 50% relative humidity, with a 12 h light: 12 h dark light cycle. Flies were reared, and maintained prior to exposure, on a cornmeal-yeast-molasses medium.

### RNA isolation, library preparation and sequencing

Following the exposure period animals were directly transferred, without anesthesia, to labeled, sterile, 2-ml screw-top tubes (Sarstedt, 72.693.005) containing 4–6 glass beads (BioSpec, 11079127, 2.7-mm diameter), and flash frozen in liquid nitrogen. Nearly all animals were alive following exposure, but those rare dead animals were removed from the exposure chambers prior to live animal collection. We immediately added 600-μl of ice-cold TRIzol Reagent (ThermoFisher, 15596018), homogenized the flies in a Mini-BeadBeater-96 (BioSpec) for 45-s, and froze the homogenate at -80C. The next day we isolated total RNA from all samples using a Direct-zol RNA Miniprep Kit (Zymo, R2050), and quantified RNA via a Qubit RNA High Sensitivity assay (Invitrogen, Q32852). Subsequently 100-ng of total RNA from each sample was used in the NEBNext Ultra II Directional RNA Library Prep Kit for Illumina, along with unique dual indexing (NEB, E7760L and E6440S), to prepare poly-A selected mRNA-sequencing libraries. Each completed library was checked on D1000 ScreenTapes (Agilent TapeStation 4150) where we observed DNA fragment distributions averaging ~ 300-bp, library concentrations were quantified using the Qubit dsDNA Broad Range kit (Invitrogen, Q32850), and finally equal amounts of each library were pooled. The 14-plex was run on a mid-output flowcell on an Illumina NextSeq 550 instrument generating 75 bp paired-end reads. Raw sequence reads are available on the NCBI Sequence Read Archive (https://www.ncbi.nlm.nih.gov/bioproject/PRJNA1030387).

### Data processing and analysis

**Fig. 1:**
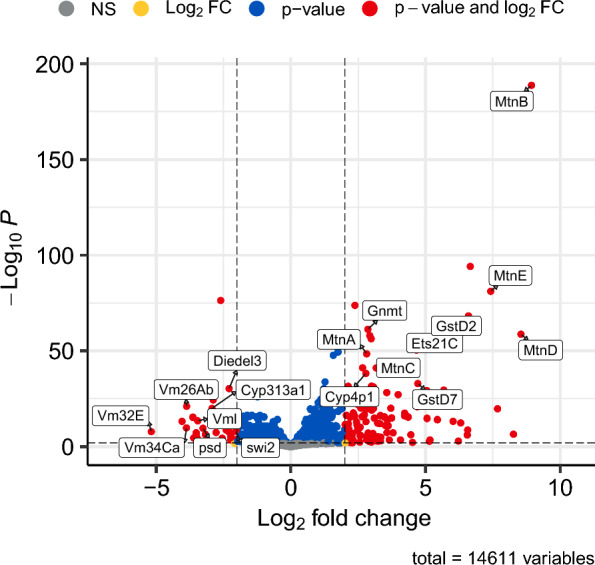
Effects of inorganic mercury exposure on gene expression. Exposure to HgCl_2_ in adult female *Drosophila* resulted in up-regulation of 119 genes and down-regulation of 31 genes compared with the control treatment. Values along the X-axis are log_2_ fold-change of the HgCl_2_/control contrast, and along the Y-axis are − log_10_ Benjamini–Hochberg FDR-adjusted *P*-values. Vertical dashed lines highlight absolute log_2_ fold-change (LFC) values > 2, horizontal dashed line indicates the significance threshold *P*_*FDR*_ < 0.01. Genes that are significant and have LFC values > 2 are shown in red, those that are significant but with LFC values < 2 are in blue, genes that are not significant but have LFC values > 2 are in yellow, and genes that are not significant and with LFC values < 2 are in grey. The top 10 upregulated and top 10 downregulated genes (ranked by *P*_*FDR*_) are annotated with gene symbols, and include all 5 metallothionein genes in *D. melanogaster* (e.g. *MtnB*), and several vitelline membrane genes (e.g. *Vm26Ab*)

Raw reads were processed with the nf-core RNA-seq pipeline v. 3.12.0 with Nextflow v. 23.04.3 [17–19]. Reads were trimmed of adapter sequences with TrimGalore v. 0.6.7 [20; Table S1], assessed for batch effects (Figure S1), and aligned to the *Drosophila melanogaster* genome assembly version 6.46 (GCF_000001215.4) with the Ensembl version 81 annotation using STAR v 2.7.10a [21] and read count quantification was performed with RSEM v. 1.3.1 [22; Table S2]. Patterns of differential gene expression between HgCl_2_ and control treatments were estimated using DESeq2 v. 1.40.2 [23] and log_2_-fold change values were adjusted with the lfcShrink function specifying the apeglm method [24; Table S3]. We classified those genes as differentially expressed that had Benjamini–Hochberg FDR-adjusted *P-*value (*P*_*FDR*_) < 0.01 and absolute log_2_ fold-change values > 2 (Fig. 1; Figure S2). Finally, we performed a gene ontology (GO) enrichment analysis with the function enrichGO in the R package clusterProfiler v 4.8.2 [25] and the *D. melanogaster* annotation provided in the R package org.Dm.eg.db v. 3.17.0 [26], focusing on the Biological Processes ontologies and specifying a *P*_*FDR*_ threshold of 0.05 (Figure S3; Table S4; Table S5). All differential expression analyses were performed in R v. 4.3.1 [27]. Plots were generated with EnhancedVolcano [28; Fig. 1], plotMDS [29; Figure S1], pheatmap [30; Figure S2], and barplot [25; Figures S3-S4].

## Results

Our libraries generated an average of 10,635,921 ± 2,254,415 (mean ± SD) 75-bp paired-end reads per sample, of which an average of 81.09% mapped to the reference genome as unique read pairs (Table S1). Initial assessment of raw gene expression data with multidimensional scaling (MDS) showed that samples clustered by treatment rather than strain genotype (Figure S1), suggesting some shifts in expression due to treatment are similar across strains. We treated the 7 strains as “replicates” in a differential expression analysis to examine such consistent changes, seeing 683 and 512 genes, respectively that are up- or downregulated at *P*_*FDR*_ < 0.01 (Table S3). By additionally applying a fold-change threshold, we identify 119 genes with significantly higher expression in HgCl_2_-treated flies (log_2_ fold-change > 2, *P*_*FDR*_ < 0.01), and 31 genes that showed significantly lower expression in HgCl_2_-treated flies (log_2_ fold-change < − 2, *P*_*FDR*_ < 0.01; Fig. 1; Figure S2; Table S2). Here, recognizing that strains are likely to exhibit differences in the response to mercury that we are unable to assess with our design, we elected to focus solely on these genes with relatively larger changes in expression.

To understand the potential functional significance of the expression changes observed we performed gene ontology (GO) term enrichment, focusing on the Biological Processes category. The terms enriched among genes showing higher expression in HgCl_2_-treated flies included response to heat and abiotic stresses, and protein folding (Figure S3; Table S4), while the terms enriched among genes with lower expression in HgCl_2_-treated flies included those related to membrane formation and stability, and egg formation (Figure S4; Table S5).

## Discussion

All five of the *Drosophila* metallothionine genes (*MtnA–MtnE*) showed increased expression following mercury exposure. These genes are expressed primarily in the digestive tract, provide essential roles in maintaining homeostasis of trace metals and heavy metal detoxification [31], and were previously shown to be up-regulated in response to copper exposure in flies from the same population we employed here [11]. Ten glutathione S-transferases (GSTs, Table S3) also showed higher expression due to HgCl_2_ treatment. Genes in this family are involved in the response to xenobiotic toxins in *Drosophila* [32], and knockdowns of GSTs—including *GstE1* which is up-regulated in our study—have been shown to increase susceptibility to the toxic effects of methylmercury in flies [33]. We also found that many heat-shock proteins showed higher expression in HgCl_2_-exposed flies. This is consistent with a prior study finding *Hsp83* was up-regulated in adults exposed to HgCl_2_ [16], although *Hsp83* was not among the set of mercury-regulated genes we identified. Interestingly, while we found that *Hsp23* increased in expression following inorganic mercury exposure in adults, Frat et al. [14] showed reduced *Hsp23* expression in larvae exposed to methylmercury. This could suggest a difference in the physiological response between larval and adult life history stages, between forms/concentrations of mercury, or variation in the response by different strains.

Fourteen of the 31 downregulated genes currently lack gene symbols (are “CG” numbers) and there is limited information available regarding their functional roles, but 7/31 are associated with oogenesis (Table S5). Notably, all individuals in our experiment were females collected at days 2–4 from mixed-sex groups, and so were likely mated. The results of our functional enrichment analysis are consistent with previous studies that showed HgCl_2_ exposure in adult *D. melanogaster* caused dose- and time-dependent atrophy of ovaries and a large reduction in egg-laying behaviors [34], and that nitrogenous toxins negatively impact egg-laying behavior in both *D. melanogaster* and *D. suzukii* [35].

In summary, we identified 119 genes that were up-regulated and 31 genes that were down-regulated in response to inorganic mercury exposure in adult female *Drosophila*. Our results recapitulate some past findings, and highlight some intriguing differences with prior studies. Given that the lines we assayed are part of a much larger set of genotyped inbred lines [10], future work can leverage the results we present here to assist in uncovering the genetic basis of variation in mercury toxicity, and help to shed light on the broader impact of exposure to this toxicant in this model system.

## Limitations

First, our study did not employ within-strain replication, so we obtained information on only the “average” strain response, and were not able to characterize genotype-to-genotype variation in the expression change driven by mercury. Such variation is highly likely to exist [36–38], and can be evaluated in future studies. Second, although there is no accepted standard inorganic mercury dosage in *Drosophila* studies—a range of concentrations have been used, from up to 100 µM [16] to 400 µM [39, 40]—the dose we employed (625 µM) is relatively high. The dose was chosen to ensure we elicited an expression response in this small pilot study, and we anticipate that experiments testing difference dosages would find some dose-dependent variation in the response. Third, although a standardized dose of inorganic mercury was provided to flies in their media, the dose actually ingested by flies was not quantified, so it is plausible that unrecognized variation in the amount of mercury consumed may have impacted gene expression. Fourth, our study included only female flies; it is clear that there is sexual dimorphism for gene expression [41] and other quantitative traits [42], so the generality of our results for understanding the response of both sexes to mercury will require further study.

## Supplementary Information

Supplementary file 1.

Supplementary file 2.

## Data Availability

The datasets generated and analyzed during the current study are available in the NCBI Sequence Read Archive, https://www.ncbi.nlm.nih.gov/bioproject/PRJNA1030387.
