# Supplementary material for: Acute exposure to mercury drives changes in gene expression in Drosophila melanogaster
Source: BMC Res Notes. 2024 Sep 30;17:279. doi: 10.1186/s13104-024-06945-y (PMC11443822; doi:10.1186/s13104-024-06945-y)
Supplement: Supplementary file 1 [file 13104_2024_6945_MOESM1_ESM.docx]

**Supplemental Figures Legends**

**Figure S1**

Multidimensional scaling plot (MDS) of raw count data for all samples. Text color represents whether samples were in the HgCl_2_ (red) or control (blue) treatment group.

**Figure S2**

Heatmap of gene expression in whole female *D. melanogaster* exposed to HgCl_2_ or control treatments. The color gradient represents variation in log_2_-transformed read counts for each of 150 genes that showed significant differential gene expression between treatments (*P_FDR_* < 0.01, |log_2_ fold-change| > 2). The column marginal color annotation reflects whether samples were in the HgCl_2_ (gray) or control (blue) treatment group.

**Figure S3**

Plot of the top 15 enriched Biological Process categories for 119 genes that exhibited higher expression in HgCl_2_-treated *D. melanogaster* (log_2_ fold-change > 2, *P_FDR_* < 0.01). Color reflects variation in significance of enrichment measured as a Benjamini–Hochberg FDR-adjusted *P-*value**.**

**Figure S4**

Plot of the top 15 enriched Biological Process categories for 31 genes that exhibited lower expression in HgCl_2_-treated *D. melanogaster* (log_2_ fold-change < -2, *P_FDR_* < 0.01). Color reflects variation in significance of enrichment measured as a Benjamini–Hochberg FDR-adjusted *P-*value**.**

**Figure S1**

**
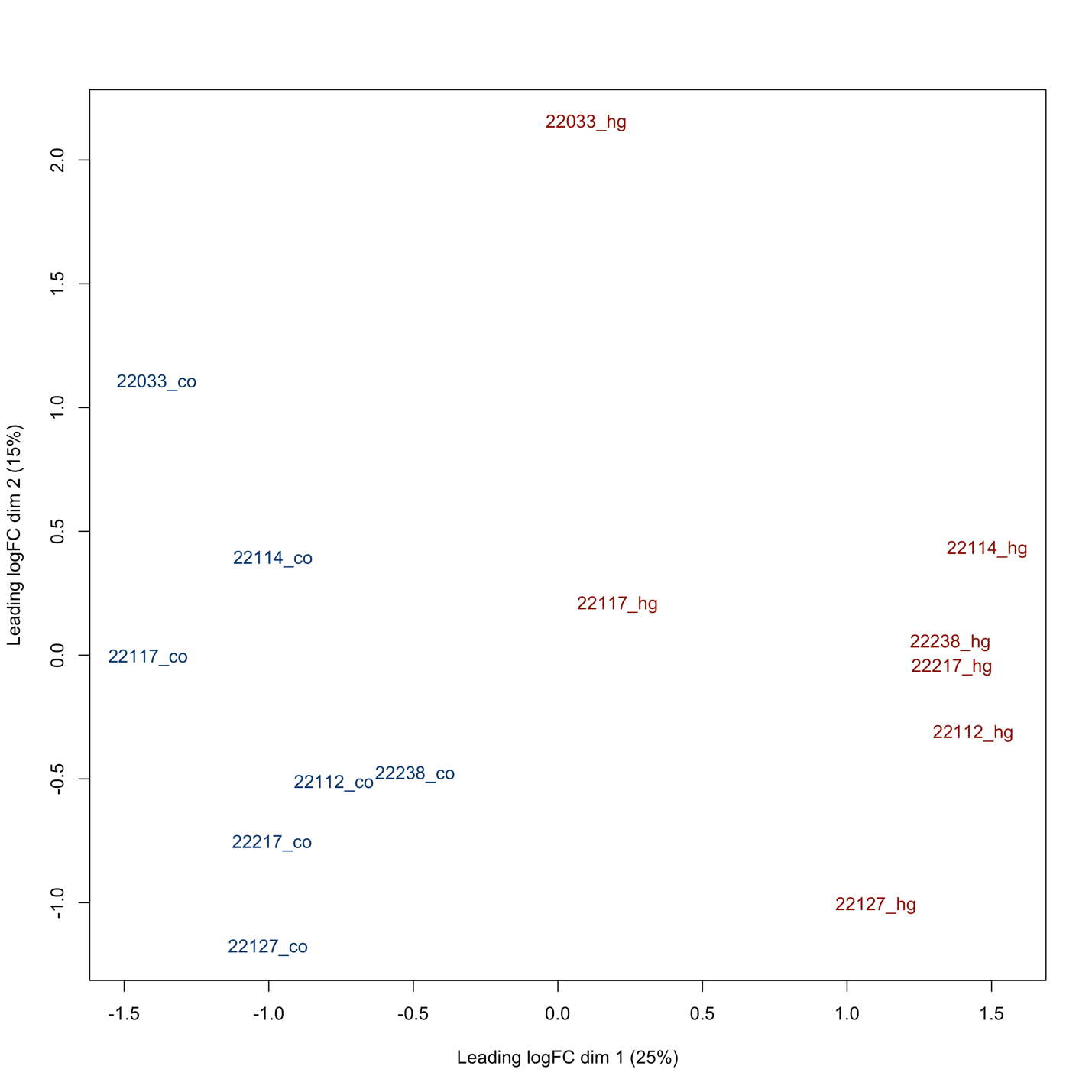
**

**Figure S2**

**
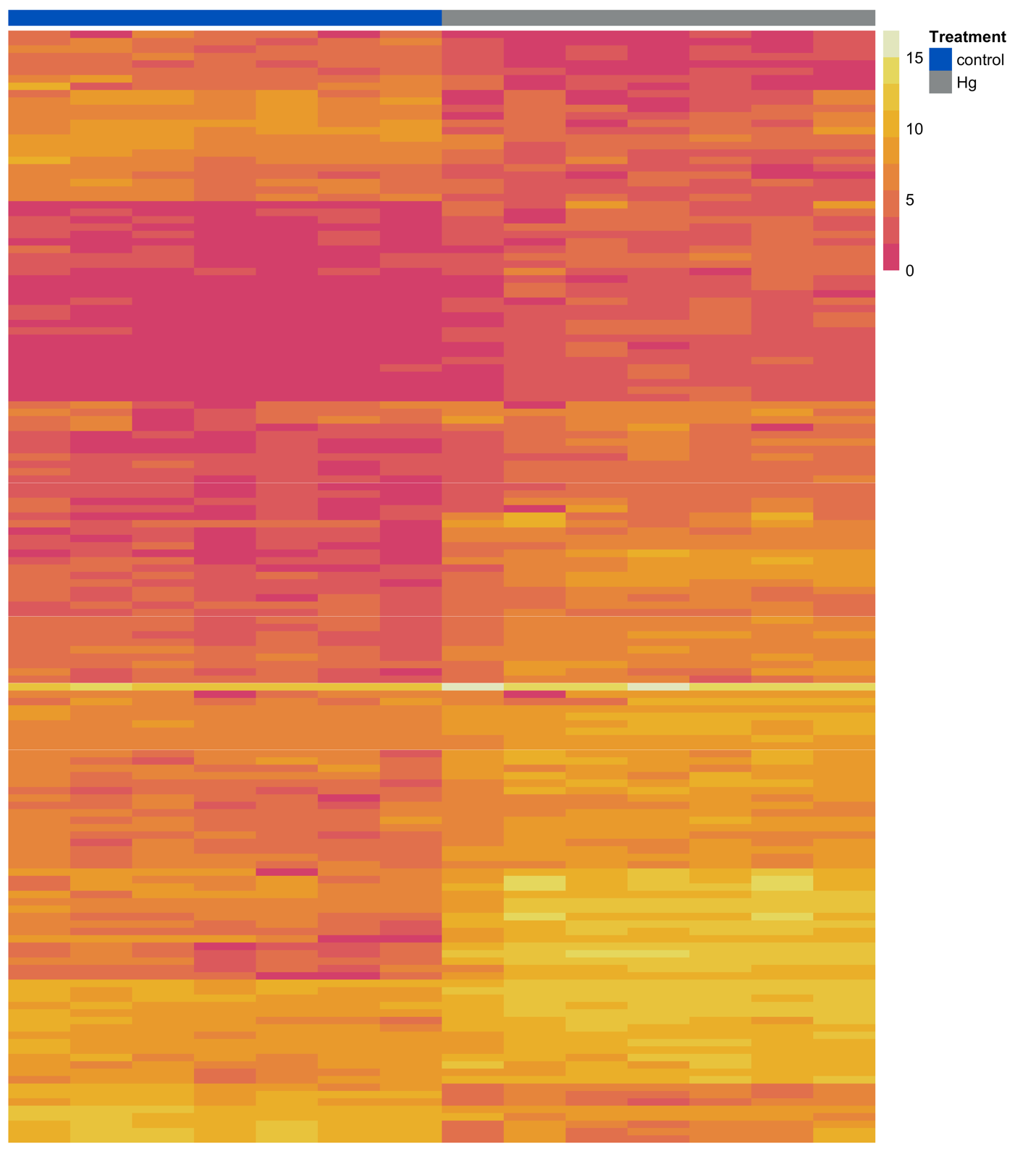
**

**Figure S3**

**
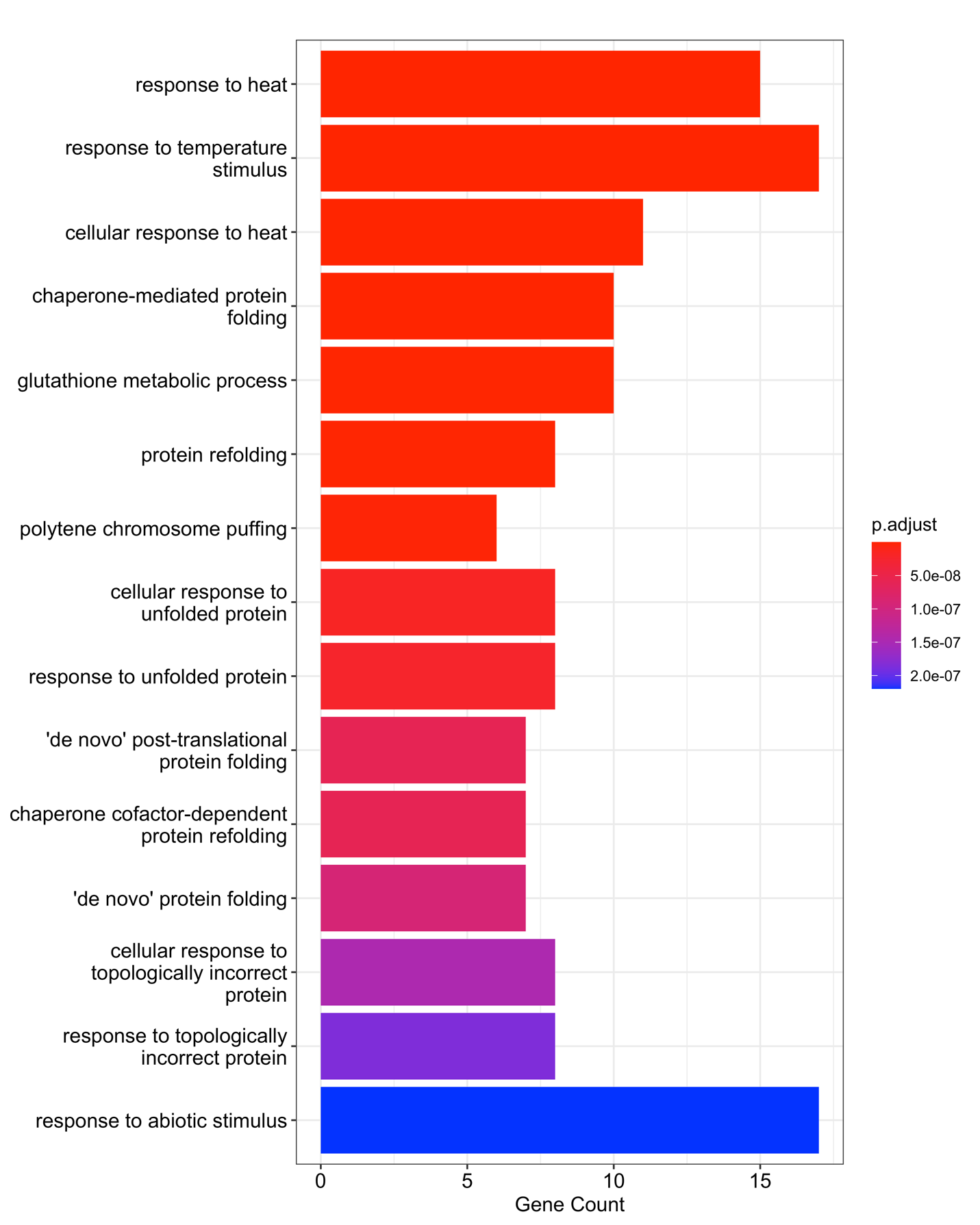
**

**Figure S4**

**
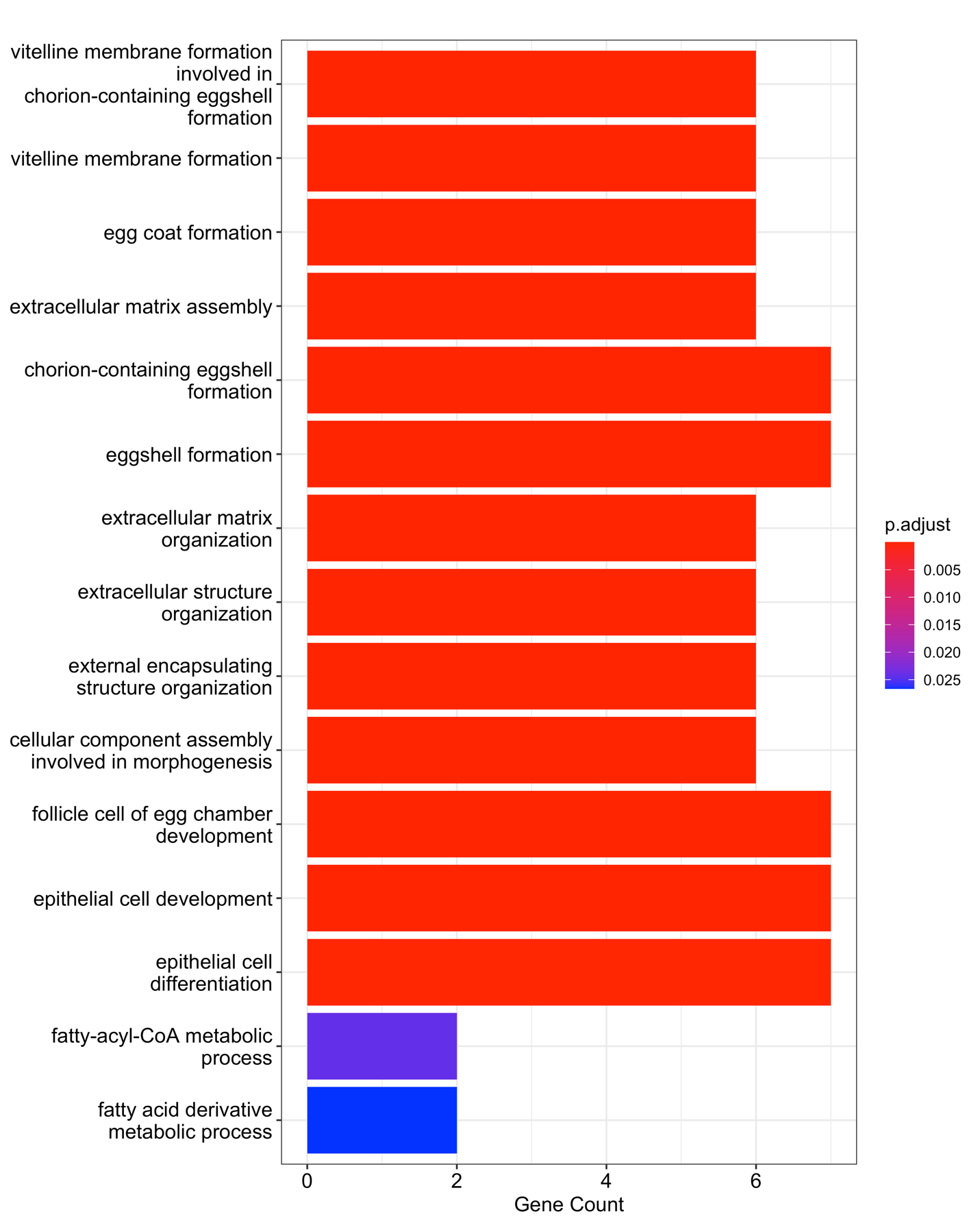
**
